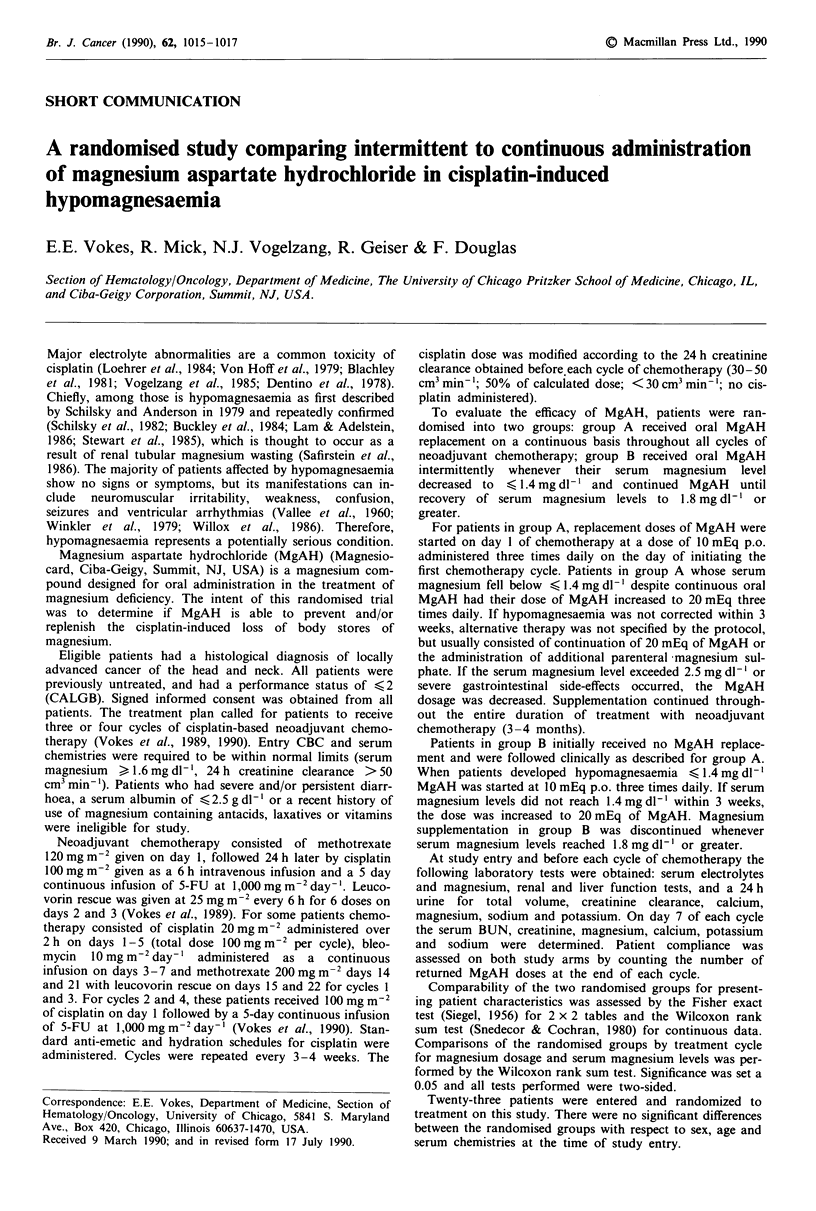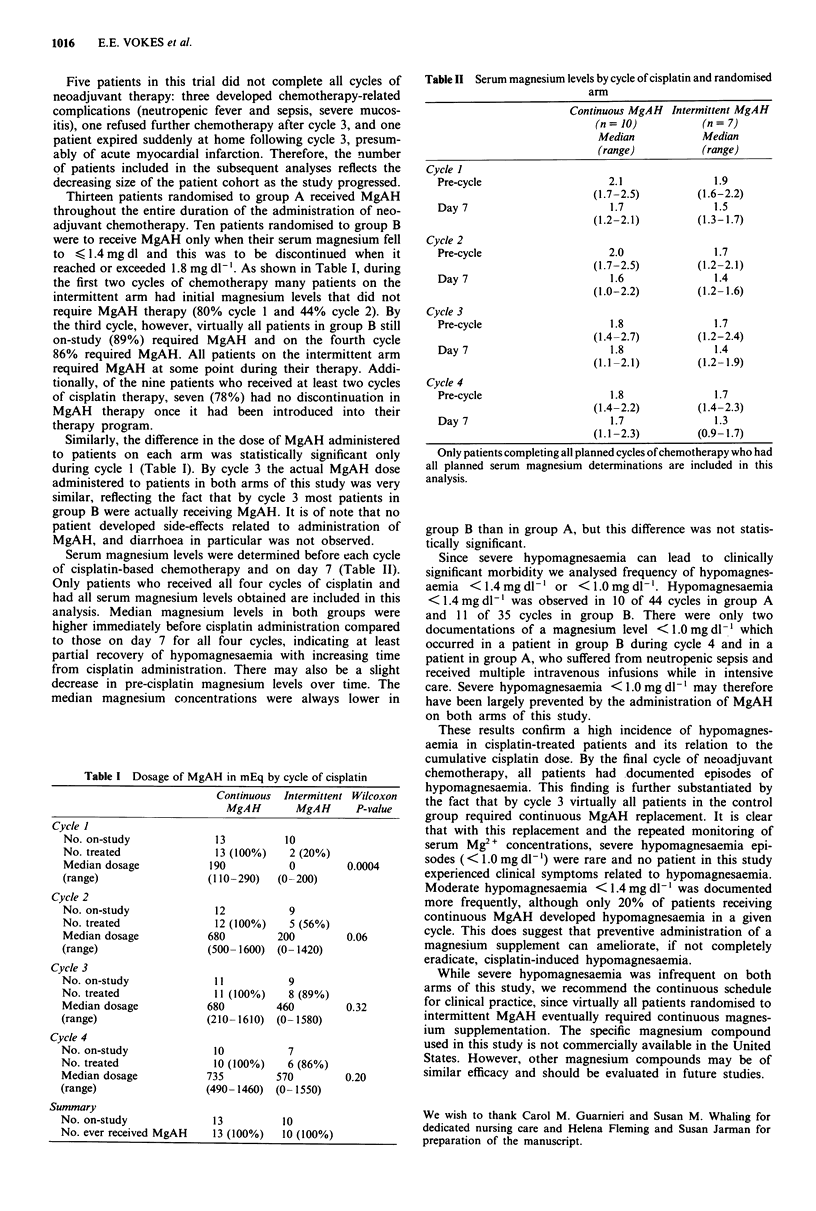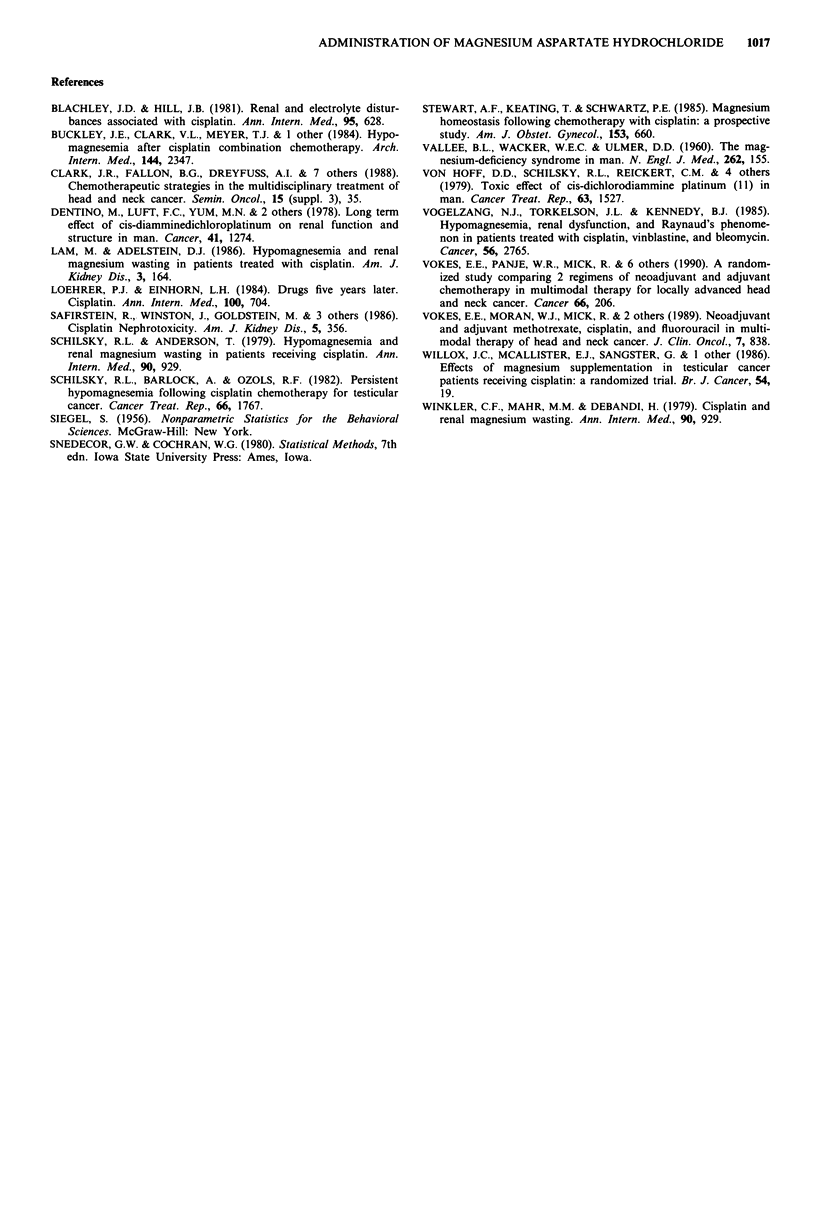# A randomised study comparing intermittent to continuous administration of magnesium aspartate hydrochloride in cisplatin-induced hypomagnesaemia.

**DOI:** 10.1038/bjc.1990.429

**Published:** 1990-12

**Authors:** E. E. Vokes, R. Mick, N. J. Vogelzang, R. Geiser, F. Douglas

**Affiliations:** Department of Medicine, University of Chicago Pritzker School of Medicine, IL.


Br. J. Cancer (1990), 62, 1015-1017 © Macmillan Press Ltd., 1990

SHORT COMMUNICATION

### A randomised study comparing intermittent to continuous administration of magnesium aspartate hydrochloride in cisplatin-induced hypomagnesaemia

E.E. Vokes, R. Mick, N.J. Vogelzang, R. Geiser & F. Douglas

*Section of Hematology/Oncology, Department of Medicine, The University of Chicago Pritzker School of Medicine, Chicago, IL, and Ciba-Geigy Corporation, Summit, NJ, USA.*

Major electrolyte abnormalities are a common toxicity of cisplatin (Loehrer *et al.*, 1984; Von Hoff *et al.*, 1979; Blachley *et al.*, 1981; Vogelzang *et al.*, 1985; Dentino *et al.*, 1978). Chiefly, among those is hypomagnesaemia as first described by Schilsky and Anderson in 1979 and repeatedly confirmed (Schilsky *et al.*, 1982; Buckley *et al.*, 1984; Lam & Adelstein, 1986; Stewart *et al.*, 1985), which is thought to occur as a result of renal tubular magnesium wasting (Safirstein *et al.*, 1986). The majority of patients affected by hypomagnesaemia show no signs or symptoms, but its manifestations can include neuromuscular irritability, weakness, confusion, seizures and ventricular arrhythmias (Vallee *et al.*, 1960; Winkler *et al.*, 1979; Willox *et al.*, 1986). Therefore, hypomagnesaemia represents a potentially serious condition.

Magnesium aspartate hydrochloride (MgAH) (Magnesiocard, Ciba-Geigy, Summit, NJ, USA) is a magnesium compound designed for oral administration in the treatment of magnesium deficiency. The intent of this randomised trial was to determine if MgAH is able to prevent and/or replenish the cisplatin-induced loss of body stores of magnesium.

Eligible patients had a histological diagnosis of locally advanced cancer of the head and neck. All patients were previously untreated, and had a performance status of $\leqslant 2$ (CALGB). Signed informed consent was obtained from all patients. The treatment plan called for patients to receive three or four cycles of cisplatin-based neoadjuvant chemotherapy (Vokes *et al.*, 1989, 1990). Entry CBC and serum chemistries were required to be within normal limits (serum magnesium $\geqslant 1.6\,mg\,dl^{-1}$, 24 h creatinine clearance $>50\,cm^3\,min^{-1}$). Patients who had severe and/or persistent diarrhoea, a serum albumin of $\leqslant 2.5\,g\,dl^{-1}$ or a recent history of use of magnesium containing antacids, laxatives or vitamins were ineligible for study.

Neoadjuvant chemotherapy consisted of methotrexate $120\,mg\,m^{-2}$ given on day 1, followed 24 h later by cisplatin $100\,mg\,m^{-2}$ given as a 6 h intravenous infusion and a 5 day continuous infusion of 5-FU at $1{,}000\,mg\,m^{-2}\,day^{-1}$. Leucovorin rescue was given at $25\,mg\,m^{-2}$ every 6 h for 6 doses on days 2 and 3 (Vokes *et al.*, 1989). For some patients chemotherapy consisted of cisplatin $20\,mg\,m^{-2}$ administered over 2 h on days 1-5 (total dose $100\,mg\,m^{-2}$ per cycle), bleomycin $10\,mg\,m^{-2}\,day^{-1}$ administered as a continuous infusion on days 3-7 and methotrexate $200\,mg\,m^{-2}$ days 14 and 21 with leucovorin rescue on days 15 and 22 for cycles 1 and 3. For cycles 2 and 4, these patients received $100\,mg\,m^{-2}$ of cisplatin on day 1 followed by a 5-day continuous infusion of 5-FU at $1{,}000\,mg\,m^{-2}\,day^{-1}$ (Vokes *et al.*, 1990). Standard anti-emetic and hydration schedules for cisplatin were administered. Cycles were repeated every 3-4 weeks. The cisplatin dose was modified according to the 24 h creatinine clearance obtained before each cycle of chemotherapy (30-50 $cm^3\,min^{-1}$; 50% of calculated dose; $<30\,cm^3\,min^{-1}$; no cisplatin administered).

To evaluate the efficacy of MgAH, patients were randomised into two groups: group A received oral MgAH replacement on a continuous basis throughout all cycles of neoadjuvant chemotherapy; group B received oral MgAH intermittently whenever their serum magnesium level decreased to $\leqslant 1.4\,mg\,dl^{-1}$ and continued MgAH until recovery of serum magnesium levels to $1.8\,mg\,dl^{-1}$ or greater.

For patients in group A, replacement doses of MgAH were started on day 1 of chemotherapy at a dose of 10 mEq p.o. administered three times daily on the day of initiating the first chemotherapy cycle. Patients in group A whose serum magnesium fell below $\leqslant 1.4\,mg\,dl^{-1}$ despite continuous oral MgAH had their dose of MgAH increased to 20 mEq three times daily. If hypomagnesaemia was not corrected within 3 weeks, alternative therapy was not specified by the protocol, but usually consisted of continuation of 20 mEq of MgAH or the administration of additional parenteral magnesium sulphate. If the serum magnesium level exceeded $2.5\,mg\,dl^{-1}$ or severe gastrointestinal side-effects occurred, the MgAH dosage was decreased. Supplementation continued throughout the entire duration of treatment with neoadjuvant chemotherapy (3-4 months).

Patients in group B initially received no MgAH replacement and were followed clinically as described for group A. When patients developed hypomagnesaemia $\leqslant 1.4\,mg\,dl^{-1}$ MgAH was started at 10 mEq p.o. three times daily. If serum magnesium levels did not reach $1.4\,mg\,dl^{-1}$ within 3 weeks, the dose was increased to 20 mEq of MgAH. Magnesium supplementation in group B was discontinued whenever serum magnesium levels reached $1.8\,mg\,dl^{-1}$ or greater.

At study entry and before each cycle of chemotherapy the following laboratory tests were obtained: serum electrolytes and magnesium, renal and liver function tests, and a 24 h urine for total volume, creatinine clearance, calcium, magnesium, sodium and potassium. On day 7 of each cycle the serum BUN, creatinine, magnesium, calcium, potassium and sodium were determined. Patient compliance was assessed on both study arms by counting the number of returned MgAH doses at the end of each cycle.

Comparability of the two randomised groups for presenting patient characteristics was assessed by the Fisher exact test (Siegel, 1956) for 2 × 2 tables and the Wilcoxon rank sum test (Snedecor & Cochran, 1980) for continuous data. Comparisons of the randomised groups by treatment cycle for magnesium dosage and serum magnesium levels was performed by the Wilcoxon rank sum test. Significance was set a 0.05 and all tests performed were two-sided.

Twenty-three patients were entered and randomized to treatment on this study. There were no significant differences between the randomised groups with respect to sex, age and serum chemistries at the time of study entry.

Correspondence: E.E. Vokes, Department of Medicine, Section of Hematology/Oncology, University of Chicago, 5841 S. Maryland Ave., Box 420, Chicago, Illinois 60637-1470, USA.
Received 9 March 1990; and in revised form 17 July 1990.

Five patients in this trial did not complete all cycles of neoadjuvant therapy: three developed chemotherapy-related complications (neutropenic fever and sepsis, severe mucositis), one refused further chemotherapy after cycle 3, and one patient expired suddenly at home following cycle 3, presumably of acute myocardial infarction. Therefore, the number of patients included in the subsequent analyses reflects the decreasing size of the patient cohort as the study progressed.

Thirteen patients randomised to group A received MgAH throughout the entire duration of the administration of neoadjuvant chemotherapy. Ten patients randomised to group B were to receive MgAH only when their serum magnesium fell to $\leqslant 1.4$ mg dl and this was to be discontinued when it reached or exceeded 1.8 mg $dl^{-1}$. As shown in Table I, during the first two cycles of chemotherapy many patients on the intermittent arm had initial magnesium levels that did not require MgAH therapy (80% cycle 1 and 44% cycle 2). By the third cycle, however, virtually all patients in group B still on-study (89%) required MgAH and on the fourth cycle 86% required MgAH. All patients on the intermittent arm required MgAH at some point during their therapy. Additionally, of the nine patients who received at least two cycles of cisplatin therapy, seven (78%) had no discontinuation in MgAH therapy once it had been introduced into their therapy program.

Similarly, the difference in the dose of MgAH administered to patients on each arm was statistically significant only during cycle 1 (Table I). By cycle 3 the actual MgAH dose administered to patients in both arms of this study was very similar, reflecting the fact that by cycle 3 most patients in group B were actually receiving MgAH. It is of note that no patient developed side-effects related to administration of MgAH, and diarrhoea in particular was not observed.

Serum magnesium levels were determined before each cycle of cisplatin-based chemotherapy and on day 7 (Table II). Only patients who received all four cycles of cisplatin and had all serum magnesium levels obtained are included in this analysis. Median magnesium levels in both groups were higher immediately before cisplatin administration compared to those on day 7 for all four cycles, indicating at least partial recovery of hypomagnesaemia with increasing time from cisplatin administration. There may also be a slight decrease in pre-cisplatin magnesium levels over time. The median magnesium concentrations were always lower in group B than in group A, but this difference was not statistically significant.

**Table I** Dosage of MgAH in mEq by cycle of cisplatin

| | *Continuous MgAH* | *Intermittent MgAH* | *Wilcoxon P-value* |
|---|---|---|---|
| *Cycle 1* | | | |
| No. on-study | 13 | 10 | |
| No. treated | 13 (100%) | 2 (20%) | |
| Median dosage | 190 | 0 | 0.0004 |
| (range) | (110–290) | (0–200) | |
| *Cycle 2* | | | |
| No. on-study | 12 | 9 | |
| No. treated | 12 (100%) | 5 (56%) | |
| Median dosage | 680 | 200 | 0.06 |
| (range) | (500–1600) | (0–1420) | |
| *Cycle 3* | | | |
| No. on-study | 11 | 9 | |
| No. treated | 11 (100%) | 8 (89%) | |
| Median dosage | 680 | 460 | 0.32 |
| (range) | (210–1610) | (0–1580) | |
| *Cycle 4* | | | |
| No. on-study | 10 | 7 | |
| No. treated | 10 (100%) | 6 (86%) | |
| Median dosage | 735 | 570 | 0.20 |
| (range) | (490–1460) | (0–1550) | |
| *Summary* | | | |
| No. on-study | 13 | 10 | |
| No. ever received MgAH | 13 (100%) | 10 (100%) | |

**Table II** Serum magnesium levels by cycle of cisplatin and randomised arm

| | *Continuous MgAH (n = 10) Median (range)* | *Intermittent MgAH (n = 7) Median (range)* |
|---|---|---|
| *Cycle 1* | | |
| Pre-cycle | 2.1 (1.7–2.5) | 1.9 (1.6–2.2) |
| Day 7 | 1.7 (1.2–2.1) | 1.5 (1.3–1.7) |
| *Cycle 2* | | |
| Pre-cycle | 2.0 (1.7–2.5) | 1.7 (1.2–2.1) |
| Day 7 | 1.6 (1.0–2.2) | 1.4 (1.2–1.6) |
| *Cycle 3* | | |
| Pre-cycle | 1.8 (1.4–2.7) | 1.7 (1.2–2.4) |
| Day 7 | 1.8 (1.1–2.1) | 1.4 (1.2–1.9) |
| *Cycle 4* | | |
| Pre-cycle | 1.8 (1.4–2.2) | 1.7 (1.4–2.3) |
| Day 7 | 1.7 (1.1–2.3) | 1.3 (0.9–1.7) |

Only patients completing all planned cycles of chemotherapy who had all planned serum magnesium determinations are included in this analysis.

Since severe hypomagnesaemia can lead to clinically significant morbidity we analysed frequency of hypomagnesaemia $< 1.4$ mg $dl^{-1}$ or $< 1.0$ mg $dl^{-1}$. Hypomagnesaemia $< 1.4$ mg $dl^{-1}$ was observed in 10 of 44 cycles in group A and 11 of 35 cycles in group B. There were only two documentations of a magnesium level $< 1.0$ mg $dl^{-1}$ which occurred in a patient in group B during cycle 4 and in a patient in group A, who suffered from neutropenic sepsis and received multiple intravenous infusions while in intensive care. Severe hypomagnesaemia $< 1.0$ mg $dl^{-1}$ may therefore have been largely prevented by the administration of MgAH on both arms of this study.

These results confirm a high incidence of hypomagnesaemia in cisplatin-treated patients and its relation to the cumulative cisplatin dose. By the final cycle of neoadjuvant chemotherapy, all patients had documented episodes of hypomagnesaemia. This finding is further substantiated by the fact that by cycle 3 virtually all patients in the control group required continuous MgAH replacement. It is clear that with this replacement and the repeated monitoring of serum $Mg^{2+}$ concentrations, severe hypomagnesaemia episodes ($< 1.0$ mg $dl^{-1}$) were rare and no patient in this study experienced clinical symptoms related to hypomagnesaemia. Moderate hypomagnesaemia $< 1.4$ mg $dl^{-1}$ was documented more frequently, although only 20% of patients receiving continuous MgAH developed hypomagnesaemia in a given cycle. This does suggest that preventive administration of a magnesium supplement can ameliorate, if not completely eradicate, cisplatin-induced hypomagnesaemia.

While severe hypomagnesaemia was infrequent on both arms of this study, we recommend the continuous schedule for clinical practice, since virtually all patients randomised to intermittent MgAH eventually required continuous magnesium supplementation. The specific magnesium compound used in this study is not commercially available in the United States. However, other magnesium compounds may be of similar efficacy and should be evaluated in future studies.

We wish to thank Carol M. Guarnieri and Susan M. Whaling for dedicated nursing care and Helena Fleming and Susan Jarman for preparation of the manuscript.

**References**

BLACHLEY, J.D. & HILL, J.B. (1981). Renal and electrolyte disturbances associated with cisplatin. *Ann. Intern. Med.*, **95**, 628.

BUCKLEY, J.E., CLARK, V.L., MEYER, T.J. & 1 other (1984). Hypomagnesemia after cisplatin combination chemotherapy. *Arch. Intern. Med.*, **144**, 2347.

CLARK, J.R., FALLON, B.G., DREYFUSS, A.I. & 7 others (1988). Chemotherapeutic strategies in the multidisciplinary treatment of head and neck cancer. *Semin. Oncol.*, **15** (suppl. 3), 35.

DENTINO, M., LUFT, F.C., YUM, M.N. & 2 others (1978). Long term effect of cis-diamminedichloroplatinum on renal function and structure in man. *Cancer*, **41**, 1274.

LAM, M. & ADELSTEIN, D.J. (1986). Hypomagnesemia and renal magnesium wasting in patients treated with cisplatin. *Am. J. Kidney Dis.*, **3**, 164.

LOEHRER, P.J. & EINHORN, L.H. (1984). Drugs five years later. Cisplatin. *Ann. Intern. Med.*, **100**, 704.

SAFIRSTEIN, R., WINSTON, J., GOLDSTEIN, M. & 3 others (1986). Cisplatin Nephrotoxicity. *Am. J. Kidney Dis.*, **5**, 356.

SCHILSKY, R.L. & ANDERSON, T. (1979). Hypomagnesemia and renal magnesium wasting in patients receiving cisplatin. *Ann. Intern. Med.*, **90**, 929.

SCHILSKY, R.L., BARLOCK, A. & OZOLS, R.F. (1982). Persistent hypomagnesemia following cisplatin chemotherapy for testicular cancer. *Cancer Treat. Rep.*, **66**, 1767.

SIEGEL, S. (1956). *Nonparametric Statistics for the Behavioral Sciences*. McGraw-Hill: New York.

SNEDECOR, G.W. & COCHRAN, W.G. (1980). *Statistical Methods*, 7th edn. Iowa State University Press: Ames, Iowa.

STEWART, A.F., KEATING, T. & SCHWARTZ, P.E. (1985). Magnesium homeostasis following chemotherapy with cisplatin: a prospective study. *Am. J. Obstet. Gynecol.*, **153**, 660.

VALLEE, B.L., WACKER, W.E.C. & ULMER, D.D. (1960). The magnesium-deficiency syndrome in man. *N. Engl. J. Med.*, **262**, 155.

VON HOFF, D.D., SCHILSKY, R.L., REICKERT, C.M. & 4 others (1979). Toxic effect of cis-dichlorodiammine platinum (11) in man. *Cancer Treat. Rep.*, **63**, 1527.

VOGELZANG, N.J., TORKELSON, J.L. & KENNEDY, B.J. (1985). Hypomagnesemia, renal dysfunction, and Raynaud's phenomenon in patients treated with cisplatin, vinblastine, and bleomycin. *Cancer*, **56**, 2765.

VOKES, E.E., PANJE, W.R., MICK, R. & 6 others (1990). A randomized study comparing 2 regimens of neoadjuvant and adjuvant chemotherapy in multimodal therapy for locally advanced head and neck cancer. *Cancer* **66**, 206.

VOKES, E.E., MORAN, W.J., MICK, R. & 2 others (1989). Neoadjuvant and adjuvant methotrexate, cisplatin, and fluorouracil in multimodal therapy of head and neck cancer. *J. Clin. Oncol.*, **7**, 838.

WILLOX, J.C., MCALLISTER, E.J., SANGSTER, G. & 1 other (1986). Effects of magnesium supplementation in testicular cancer patients receiving cisplatin: a randomized trial. *Br. J. Cancer*, **54**, 19.

WINKLER, C.F., MAHR, M.M. & DEBANDI, H. (1979). Cisplatin and renal magnesium wasting. *Ann. Intern. Med.*, **90**, 929.